# An Overview of Hormone-Sensitive Lipase (HSL)

**DOI:** 10.1155/2022/1964684

**Published:** 2022-12-08

**Authors:** Arwa R. Althaher

**Affiliations:** Department of Pharmacy, Al-Zaytoonah University of Jordan, Amman 11733, Jordan

## Abstract

Hormone-sensitive lipase (HSL) is a pivotal enzyme that mediates triglyceride hydrolysis to provide free fatty acids and glycerol in adipocytes in a hormonally controlled lipolysis process. Elevated plasma-free fatty acids were accompanied by insulin resistance, type-2 diabetes, and obesity. Inhibition of lipolysis through HSL inhibition may provide a mechanism to prevent the accumulation of free fatty acids and to improve the affectability of insulin and blood glucose handling in type II diabetes. The published studies that examine the structure, regulation, and function of HSL and major inhibitors were reviewed in this paper.

## 1. Introduction

Hormone-sensitive lipase (HSL) is an intracellular lipase responsible for releasing free fatty acids (FFAS) from adipose tissue into circulation as a significant energy source for most tissues by catalyzing the hydrolysis of wide-ranging substrates like triacylglycerol, diacylglycerol, monoacylglycerol, cholesteryl ester, and retinyl ester, as well as other lipid substrates [1]. HSL is highly expressed in adipose tissue in addition to a variety of other tissues such as the adrenal ovary, testis, and pancreas and in a lesser amount in skeletal muscles, cardiac muscles, and macrophages [2].

The activity of HSL is tightly regulated by energy-demand-based neuronal and hormonal action and is mediated by protein kinase A-dependent reversible cAMP serine phosphorylation [3]. HSL is activated by cAMP-dependent protein kinase A in response to adrenergic/noradrenergic stimulation [4]. HSL phosphorylation leads to increased hydrolytic activity, translocation of HSL from the cytosol to the lipid droplet surface, and hydrolysis of stored triacylglycerols [5]. Moreover, HSL is inhibited by insulin-stimulating adipocytes, resulting in decreased excretion of free fatty acids and glycerol [6].

In addition to its prominent role in FFA mobilization from adipose tissue, HSL promotes hydrolysis of a variety of substrates containing ester linkages, which include cholesteryl esters important in steroidogenic tissues to provide cholesterol for steroid hormone production, retinyl esters, and other lipid-related substrates [4, 7].

Despite its fundamental physiological importance, an oversupply of fatty acids (FAs) is highly detrimental. Elevated levels of unesterified FAs disrupt biological membrane integrity, alter cellular acid-base homeostasis, and form damaging bioactive lipids. These effects, in turn, impair membrane function and induce endoplasmic reticulum stress, mitochondrial dysfunction, inflammation, and cell death. In summary, these detrimental effects are grouped under lipotoxicity [8]. For protection, the cells can detoxify unesterified FAs by esterifying them with glycerol to generate triglycerides and transiently store them in adipose tissues [8].

Furthermore, lack of and alternations in hormone-sensitive lipase expression in different cell types cause high levels of triglycerides, which lead to profound effects on whole-body homeostasis, including alterations in insulin signaling and dysregulation in lipid hydrolysis (dyslipidemia) [9]. These defects are directly linked with human disorders, such as obesity, insulin resistance, type 2 diabetes, and hyperlipidemia [10], that induce enlargement of adipose tissue mass and reduce insulin sensitivity, which could cause dysregulation of lipolysis.

Due to their inability to dissolve in water, lipids like cholesterol and triglycerides must be carried in the bloodstream in combination with proteins (lipoproteins). Apolipoproteins, phospholipids, and free cholesterol surround a central core of cholesterol esters and triglycerides in lipoproteins, allowing the synthesis and function of these complex particles. Based on size, lipid composition, and apolipoproteins, plasma lipoproteins can be classified into seven groups: chylomicrons, chylomicron remnants, very low-density lipoproteins (VLDL), intermediate-density lipoproteins (IDL), low-density lipoproteins (LDL), high-density lipoproteins (HDL), and lipoprotein (a) (Lp (a)). HDL is antiatherogenic, but chylomicron remnants, VLDL, IDL, LDL, and Lp (a), are all proatherogenic [11].

On the other hand, elevated plasma free fatty acids due to the elevation in the lipolytic rate (high HSL activity) could damage lipid profiles by improving the‏ production of very-low-density lipoprotein (VLDL) by the liver, pushing toward dyslipidemia [12, 13], decrease insulin sensitivity in the tissue, and cause insulin resistance development, type 2 diabetes, and other metabolic abnormalities [14].

Additionally, many synthetic and natural HSL inhibitors have been identified [15–23]. These inhibitors provide a novel therapeutic tool that targets dyslipidemia by controlling lipid hydrolysis and reversing insulin resistance and other obesity-related metabolic problems.

Many research efforts have focused on understanding this enzyme's activity, regulation, expression, function, and inhibitors. This review briefly reported HSL's structure and biochemical properties, in addition to its regulation, deficiency, physiological function, and main natural and synthetic inhibitors.

## 2. Structural and Biochemical Properties

The human HSL is a single polypeptide of 786 residues with a molecular mass of 85.5 kDa, encoded by the *LIPE* gene located on chromosome 19 and encoded by nine exons spanning approximately 11 kb of DNA [24]. The promoter of the HSL gene has no TATA or CCAAT box and has consensus sequences such as a GC-rich region, an AT-rich region, and an initiator region [25].

Adipocyte HSL (major isoform) has a single polypeptide of 775 residues (MW∼84 kDa) composed of three domains: N-terminal binding domain, the C-terminal catalytic domain, and the regulatory domain [24, 26, 27]. The N-terminal domain is encoded by exons 1–4 (∼300 residues), mediating protein-to-protein and protein-to-lipid interactions [28]. The C-terminal domain is encoded by exons 5–9 (about 440 residues). It has the catalytic triad composed of Ser-Asp-His with several phosphorylation sites [29] and *α*/*β*-hydrolase fold, which has a highly conserved Ser residue in the Gly-x-Ser-x-Gly sequence that adjusts the catalytic site [30]. The regulatory domain is composed of ∼150 amino acids, which is the main target of protein kinase A [31]. The catalytic site (Ser423) is encoded by exon 6, and the lipid-binding region in the C-terminal is encoded by exon 9. While exon 8 encodes two phosphorylation sites, the first site (Ser551) controls cyclic AMP-mediated activity, and the second site (Ser553) is phosphorylated by 5′-AMP-activated protein kinase [25, 32].

At least three isoforms of HSL were reported in mammalian species, ranging in size from 84 to 130 kDa [33]. Two isoforms of HSL were discovered in the human testis (the HSL test). The first one was like adipose HSL (84 kDa), and the second appeared bigger than HSL adi and was around 116 kDa and had 1076 residues [34]. More studies of the HSL genes show that another exon (exon T), 16 kb upstream of exon 1, encodes nearly 301 residues in addition to those encoded by exons 1–9 [35].

Pancreatic *β* cells have a slightly larger HSL isoform (89 kDa) than the adipose form [36]. *β* Cells may have a specific exon (exon A) ∼13 kB upstream of exon 1 [35, 36] in addition to exons 1–9.

There are several other exons (B, C, and D) located upstream of exon 1 in the HSL gene [34, 37] and T2 [38] but only exons A and T1 contain a coding sequence.

Only the Gly-x-Ser-x-Gly motif for the active site serine is recognized when comparing the HSL primary sequence with other mammalian lipoprotein lipases family (lipoprotein lipase, pancreatic lipase, hepatic lipase, and others) [32]. HSL shares sequence homology with microbial lipases and esterases, especially Moraxella TA144 lipase 2 [25].

HSL is a multifunctional enzyme with extensive substrate specificity; it can catalyze the hydrolysis of triacylglycerol, diacylglycerol, monoacylglycerol, cholesteryl esters, cholesteryl esters of steroid hormones, and retinyl esters in adipose tissue, as well as water-soluble substrates [39, 40]. HSL does not have phospholipase activity like many other lipases.

The activity of HSL against diacylglycerol is about 10-fold and 5-fold higher than the activity against triacylglycerol and monoacylglycerol, respectively. In contrast, the activity against cholesteryl esters is nearly twice that of triacylglycerol [41].

## 3. Regulation of HSL and Adipocyte Lipolysis

The fundamental roles of adipocytes in mammals include lipid mobilization and storage. Over 90% of lipids are stored as TAG and account for about 80% of the weight of the entire adipose tissue [42]. Nonesterified fatty acids (NEFA) are the primary secretory byproducts of adipose tissue [42]. They are produced by the lipolysis of stored TAG, which involves three main phases and at least three different lipases and is controlled by both adipocyte and nonadipocyte factors [43]. Therefore, the classic lipolytic pathway encompasses the three following consecutive steps: (i) TAG hydrolyzation via ATGL to generate fatty acids and diacylglycerol (DAG) [43]; (ii) subsequently, HSL catalyzes the hydrolysis of DAG to monoacylglycerol (MAG) and fatty acids [44]; and (iii) monoacylglycerol lipase (MGL) is needed to finalize the hydrolysis of MAG into one fatty acid and glycerol [43]. ATGL is commonly known as a lipase that initiates the degradation of TAG to generate DAG [42]. ATGL is a 54 kDa TAG hydrolase, also called phospholipase A2*ξ* or desnutrin, which belongs to the family of patatin-like phospholipase domain-containing proteins (PNPLA) with specificity for TAG as a substrate. ATGL is highly expressed in adipose tissue, and its expression increases significantly during adipocyte differentiation [42].

HSL, a cytoplasmic protein with demonstrated activity against various substrates, including TAG, DAG, cholesteryl esters, and retinyl esters, is implicated as a rate-limiting enzyme in the early stages of the lipolysis process [42].

ATGL exhibits 10-fold higher substrate specificity for TAG than DAG, selectively enabling the first step of TAG hydrolysis, leading to DAG and fatty acid formation. A critical step in activating lipolysis involves the translocation of HSL from the cytoplasmic side to the surface of lipid droplets. Upon lipolytic stimulation, HSL translocates from the cytosol to the surface of lipid droplets and interacts with perilipin 1 and neutral lipids. Strikingly, adipocytes lacking perilipin-1 cannot translocate HSL to lipid droplets after cAMP increases [42, 43].

The expression profile of HSL mirrors that of ATGL, as both enzymes hydrolyze TAG cooperatively, thus sharing some regulatory properties but with different mechanisms of enzyme control [45]. Although *β*-adrenergic stimulation exerts ATGL regulation primarily through comparative gene identification-58 (CGI-58) recruitment, HSL forms a significant target for PKA-catalyzed phosphorylation [42].

The last step of lipolysis is catalyzed with the aid of MGL, which is constitutively expressed in adipose tissue and has no affinity for DAG, TAG, or cholesteryl esters [44]. The enzymatic activity of MGL is needed during the last hydrolysis of the 2-monoacylglycerols produced with the aid of HSL activation.

HSL can be regulated by reversible phosphorylation; the evidence shows that HSL in adipocytes could be phosphorylated and activated by the cAMP-dependent protein kinase (PKA) in response to catabolic hormones such as corticotropin-releasing hormone, epinephrine, norepinephrine, and glucagon. Stimulation of beta-adrenergic receptors leads to the activation cAMP/protein kinase A pathway [4], which mediates phosphorylation and activation of HSL and translocation from the cytosol to the surface of the lipid droplet [46, 47].

Insulin counteracts the stimulation of lipolysis and promotes fatty acid storage as triacylglycerol. Insulin regulates adipocyte glucose uptake and triggers fatty acid transport protein translocation and fatty acid uptake by fat cells [6, 48, 49]. The binding of insulin to its specific cell-surface receptor results in tyrosine phosphorylation and activation of the insulin receptor, interaction with the insulin receptor substrates (IRS-1 and IRS-2), and activation of the phosphatidyl inositol 3-kinase (PI3K) complex [6, 48]. Insulin potently inhibits basal and catecholamine-induced lipolysis via phosphorylation (through a PKB/Akt-dependent action) and activation of phosphodiesterase-3B (PDE-3B). The phosphodiesterase catalyzes the breakdown of cAMP to its inactive form, lowering cAMP levels, reducing PKA activation, and preventing HSL stimulation. Insulin can also restrain lipolysis via phosphorylation of the regulatory subunit of protein phosphatase-1 (PP-1). When activated, it rapidly dephosphorylates HSL and deactivates it, thus decreasing the lipolytic rate [50–53].

Lipolysis is regulated using HSL and other proteins that target reversible phosphorylation/activation through beta-adrenergic and insulin action [49].

Numerous proteins are involved in the regulation of lipolysis; perilipin 1A is a lipid droplet coat protein in mature adipocytes that regulates the release of fatty acids and glycerol from TG in the lipid droplet [54]. Perilipin 1A creates a barrier at the droplet surface, leading to reduced lipolysis. The barrier of perilipin must be removed to increase lipolysis through phosphorylation mediated by PKA or by reducing expression [55, 56].

Beta-adrenergic stimulation causes PKA-mediated phosphorylation for both HSL and perilipin 1A. After phosphorylation, HSL can translocate from the cytosol to the lipid droplet surface, while perilipin redistributes away from the lipid droplet, allowing for phosphorylated HSL to bind to the lipid droplet and induce lipolysis [43, 47].

In addition to the previous mechanism, two novel proteins have been identified and are involved in lipolysis. (1) HSL specifically interacts with adipocyte lipid-binding protein (ALBP), a member of a family of intracellular lipid-binding proteins that bind fatty acids, retinoids, and other hydrophobic ligands [57]. ALBP and HSL form a lipolytic complex that enhances the hydrolytic activity of HSL through physical interactions between HSL and ALBP. The ALBP sequesters fatty acids and prevents product inhibition [58]. (2) Lipotransin, another protein, interacts with HSL at the lipid droplet surface and anchors it [59].

## 4. HSL Deficiency

Adipose tissue (the most prominent energy reservoir) regulates whole-body energy and glucose homeostasis [60]. Hence, any alterations in lipid metabolism are strongly interconnected with metabolic disorders and have profound consequences for homeostasis [61].

In the white adipose tissue, excess nutrients are deposited as triglycerides (TGs), and during energy demand, nonesterified fatty acids are released into the bloodstream after hydrolyzing TGs. Fatty acids are used for energy production, membrane lipid synthesis, and signaling [62].

Lipolysis is a highly controlled process of hydrolyzing TG involving a variety of cell-surface receptors, neurotransmitters, hormones, and paracrine factors [7, 8].

Hydrolyzing of triglycerides (TGs) occurs by a series of enzymes: adipose triglyceride lipase (ATGL), the major TG lipase in humans and mice, hormone-sensitive lipase (HSL), and monoglyceride lipase (MGL). HSL and MGL hydrolyze diglycerides (DGs) and monoglycerides (MGs), respectively [63].

The importance of HSL-mediated lipolysis in cellular signaling processes was displayed by [8, 12, 63] and others. Human HSL mutation causes a reduction in the lipid storage capacity (due to decreased PPAR-*γ* signaling); also, excessive circulating fatty acids lead to (ectopic fat accumulation) deposition in nonadipose tissues such as the liver and skeletal muscle and the development of insulin resistance [64].

The first clinical description of individuals with a frameshift mutation (deletion of 19 bp in the last exon results in the addition of 86 amino acids to the C terminus of the protein) in the LIPE gene encoding HSL was reported by Albert et al. [9]. This mutation causes a reduction in the abundance of HSL protein in adipose carriers because of decreases in enzyme synthesis or increases, in turn, over [9].

Hydrolysis of diglycerides (DGs) and retinyl esters (REs) by HSL produces lipophilic ligand(s) that stimulate peroxisome proliferator-activated receptor-*γ* (PPAR-*γ*) and/or retinoid-Xreceptor-a (RXRa), function as heterodimers, and act as transcription factors that bind to a DNA sequence known as peroxisome proliferator response elements (PPREs) in the promoter of target genes, which leads to adipogenesis and lipid synthesis [65].

As a result of HSL deficiency, peroxisome proliferator-activated receptor (PPAR)-*γ* signaling is lessened in adipose tissue, which causes a decrease in adipogenesis and lipid synthesis. The capacity of adipose tissue for the storage of lipids is limited; also, circulating fatty acids (FAs) are relocated to the liver, leading to ectopic lipid deposition and metabolic dysfunctions such as dyslipidemia and diabetes [12, 63].

## 5. Physiological Functions in Other Tissues

### 5.1. Muscles

Hormone-sensitive lipase (∼84 kDa) is expressed in cardiac and skeletal muscles [6]. Like the regulation of lipolysis in the adipose tissue, HSL activity in both muscles is stimulated by catecholamines via *β*-adrenergic receptors and cyclic AMP [6].

In the skeletal muscle, HSL is the rate-limiting enzyme for intra-myocellular triacylglycerol (IMTG) hydrolysis during contractions [66].

Alsted et al. reported that at least 98% of the total TG lipase in the mouse muscle are ATGL and HSL, with slight importance of other lipases for breaking IMTG. *In vitro* measurements the breakdown of IMTG during contractions in isolated skeletal muscles revealed a decrease in the hydrolysis of IMTG in rat muscles in both pharmacological inhibitions of HSL and HSL knockout mice [67].

Furthermore, another study showed that AMPK activation during moderate exercise did not impair HSL activity in human skeletal muscle in vivo, consistent with its role as a fuel supplier rather than a fuel inhibitor [68].

Overexpression of HSL in transgenic mice prevents the accumulation of cardiac triglycerides and inhibits myocardial steatosis and fibrosis [69], in addition to altering the expression of cardiac genes for fatty acid oxidation, transcription factors, signaling molecules, and cytoskeletal proteins [70].

HSL in cardiac and skeletal muscle plays a vital role in controlling the accumulation of triglycerides and could be used as a therapeutic target for regulating diabetic cardiomyopathy [70].

### 5.2. Adrenal

Hormone-sensitive lipase (HSL) is a strategic regulator of cholesterol metabolism in steroidogenic tissues (adrenals, ovaries, and testes) [71]. The adrenal gland is responsible for neutral cholesteryl ester hydrolase activity by hydrolyzing intracellular cholesteryl esters and making unesterified cholesterol accessible for steroid hormone production [72].

HSL-deficient adrenal cells exhibited a marked accumulation of lipid droplets in both the zona glomerulosa and the zona fasciculate [71]. Moreover, there is a reduction in the production of steroids in adrenal cells due to the inability to utilize cholesteryl esters [72].

### 5.3. Testis

The testicular form of HSL tes is expressed in several forms of HSL, with sizes ranging from 26 to 130 kDa), in both rodents and humans [73, 74]. HSLtes contains a larger form of 3.9 kb mRNA (in both humans and rats) due to a testis-specific exon (T exon) 15.5 kB upstream of exon 1 that encodes a protein of 120–130 kDa and has a unique NH_2_-terminal domain that produces an additional 301 amino acids in addition to the 775 amino acids common to all forms of HSL (the normal adipose form) [35]. Thus, the 3.9 kb HSLtes mRNA is translated into 1068 amino acids in rats and 1076 amino acids in proteins in humans [35]. The other form appears only in humans with 3.3 kb mRNA due to a second testis-specific exon ∼12 kb upstream of exon 1 that encodes a protein of 88 kDa like adipocyte HSL [38, 75]. In comparison, other testicular forms may arise from post-transcriptional modifications.

In the human testis, HSL is expressed in seminiferous tubules and Leydig cells [38], whereas in the rat, HSL is expressed only in the seminiferous tubules (Sertoli and spermatogenic cells) [73, 76].

The role of HSL in the testis was studied using HSL (-/-) deficient mice [77, 78]. The results showed an increase in the accumulation of testicular cholesteryl ester by 2–4 folds and DAG [77, 78], alterations in spermatid maturation and oligospermia [76], and severe morphological abnormalities in the sperm [76], which trigger male sterility.

There is a relationship between cholesteryl ester hydrolase (CEH) activity, cholesteryl ester level, and fertility. The CEH activity mediated by HSLtes in haploid germ cells is thus necessary for spermatogenesis. Deficiency in testicular HSL causes a lowering in the CEH activity and accumulation of cholesteryl esters and confers male sterility with a significant defect in spermatogenesis [74].

### 5.4. Islets

HSL is also expressed in the pancreatic islets (89 kDa) and is slightly larger than the adipocyte form (85 kDa) and smaller than HSLtes form (120 kDa). The larger form of 3.1 kb mRNA is due to exon-A ∼13 kB upstream of exon 1, containing an additional 43 amino acids N-terminal compared to the adipocyte form [36, 79]. HSL is localized in the secretory granules of *β* cells, and some HSL is also observed in *α* cells [36].

The role of HSL in *β* cells was assessed by incubating *β* cells with high glucose concentrations for 16 and 32 h, which induced HSL protein expression twofold, with no effect between 4 and 8 h of incubation [80].

HSL might be essential in insulin secretion [81]. Obese ob/ob mice were used as an animal model of noninsulin-dependent diabetes mellitus, displaying hyperglycemia, hyperinsulinemia, and obesity. Long treatment of obese mice with leptin showed decreased islet triglyceride content, improvement in insulin secretion, and increased expression of islet HSL [81, 82].

## 6. Synthetic and Natural Inhibitors of HSL

### 6.1. Synthetic Inhibitors

Free fatty acids (FFAs) are essential for maintaining energy homeostasis, but an increase in plasma FFA levels is linked to obesity, insulin resistance, type II diabetes, and neuroinflammation [83, 84].

These conditions induce the enlargement of adipose tissue mass and inhibit insulin's antilipolytic activity, which will additionally increase the rate of FFA release into the circulation, which is linked to the dysregulation of lipolysis. As well as an increase in FFA level, this boosts the production of very low-density lipoprotein (VLDL) in the hepatic cells, which is one of the leading causes of dyslipidemia [13].

Targeting FFA metabolism may provide a new therapeutic method for reducing FFA levels in plasma and peripheral insulin resistance in type 2 diabetes. Currently, HSL inhibitors are evolving rapidly for designing drugs and using them to treat dyslipidemia and insulin resistance by controlling lipolysis. Various sources of HSL inhibitors have been identified from natural resources (plants and microbes) or artificially synthesized [85, 86].

Several inhibitors of cholesterol esterase may also display activity against HSL [16, 18, 87]. Methyl-phenyl-carbamoyl-triazoles are a selective inhibitor against acetylcholine esterase activity. They do not inhibit other hydrolases such as hepatic lipase, lipoprotein lipase, pancreatic lipase, or butyrylcholine esterase, with IC_50_ values ranging from >5 *μ*M to <50 nM [87]. Carbazates revealed *in vitro* inhibitory effects (nanomolar potency) on HSL activity as well as inhibition of cholinesterase [16], in addition to a series of (5-(2H)-isoxazolyl) urease that were established as HSL inhibitors [18].

Those HSL inhibitors have been reported with different inhibition mechanisms. Most of them are pseudosubstrate irreversible inhibitors that exhibit potent inhibitory activity and an antilipolytic effect *in vivo*. In addition, they contain a reactive material such as urea or carbamate, which could cause unpredicted toxicity [87].

Another group of HSL inhibitors was reported as selective and reversible inhibitors. A series of aryl and heteroaryl boronic acids (2-Benzyloxy-5-fluorophenyl), boronic acid (2-benzyloxy-5-chlorophenyl), boronic acid, and 5-bromothiophene-2-boronic acid were evaluated *in vitro* to study the inhibitory effect against HSL. The most potent inhibitory effect was 5-bromothiophene-2-boronic acid with (IC_50_ = 140, 17, and 350 nM, respectively) [19].

Ogiyama et al. identified the boronated compound (compound 1) that exerted‏ HSL inhibitory *in vitro* activity with an IC_50_ value of 7 nM with high selectivity against cholinesterases and an *in vivo* antilipolytic effect after oral administration at 3 mg/kg in rats [88].

Furthermore, boronate compounds form reactive metabolites that are covalently attached to macromolecules such as proteins and DNA, which cause organ toxicity and carcinogenesis [89]. Consequently, reducing the ability to form reactive metabolites is required.

On the other hand, to reduce the bioactivation potential of compound 1, a series of modifications were done until compound 24b was identified, which exhibited potent HSL inhibitory activity (IC_50_ = 2 nM) with a significantly reduced bioactivation potential. In rats, oral administration of compound 24b at 3 mg/kg exhibited an antilipolytic effect [22].

Ben Ali et al. selected two compounds as HSL inhibitors: 5-methoxy-3-(3-phenoxyphenyl)-1, 3, 4-oxadiazol-2(3H)-one (compound 7600) and 5-methoxy-3-(3-methyl-4-phenylacetamidophenyl)-1, 3, 4-oxadiazol-2(3H)-one (compound 9368). The enzyme was incubated with each inhibitor alone in an aqueous medium. HSL was inhibited after incubation with compound 7600 and compound 9368. The reactivation of HSL was detected in the presence of a lipid substrate when incubating with compound 7600, while the reactivation process was scarcely observed when incubating HSL with compound 9368 [90].

The inhibition mechanism by compound 7600 includes a nucleophilic attack by the hydroxy group of the catalytic Ser of the enzyme on the carbon atom of the carbonyl moiety of the oxadiazolone ring of the inhibitor, leading to the formation of a covalent enzyme-inhibitor intermediate. After that, this intermediate was hydrolyzed in H_2_O, releasing an active form of HSL, an oxadiazolone decomposition product, and carbon dioxide [90].

Moreover, HSL and MGL are vital lipases involved in lipolysis and the degradation of triglycerides into FFA and glycerol [91]. Novel lipase inhibitors against MGL and HSL were screened from commercial agrochemicals. Quinclorac, a safe herbicide, showed antilipase activity *in vitro* against rat epididymal lipase (nanomolar IC_50_) and *in vivo* significantly decreased blood glycerol levels after acute exposure (150 mg/kg) and multiple dosing (50 or 25 mg/kg) (*p* < 0.05) [92].

### 6.2. Natural Inhibitors

Bustanji et al. reported the dual effects of rosemary as hypoglycemic andhypolipidemic. *In vitro* administration of *Rosmarinus officinalis* L. (rosemary) extract and purified compounds found in rosemary (rosmarinic acid (RA), chlorogenic acid (CA), caffeic acid (CAA), and gallic acid (GA)) showed inhibitory effect on both HSL and pancreatic lipase (PL) dependent on concentrations with different capacities [21].

Rosemary extract had an IC_50_ (13.8 *μ*g/mL) for PL lesser than the IC_50_ for HSL (95.2 *μ*g/mL). While in purified, tested compounds (RA, CA, CAA, and GA), GA is the most powerful (IC_50_ 10.1 and 14.5 for PL and HSL, respectively).

Another study of twelve plant extracts showed inhibition of HSL activity in a dose-dependent manner [21]. Three out of twelve extracts, *Malva nicaeensis*, *Haplophyllum buxbaumii*, and *Anchusa italica*, exhibited more than 30% inhibition against HSL activity, with IC_50_ (51.1, 101.3, and 132.8 *μ*g/ml), respectively. The other 9 plant extracts showed moderate to weak activities: *Cinnamomum verum* (25.8% of inhibition), *Linum pubescens* (25.5%), *Hypecoum dimidiatum* (25.0%), *Varthemia iphionoides* (23.6%), *Convolvulus althaeoides* (22.6%), *Paronychia argentea* (20.0%), *Eryngium creticum* (18.7%), *Majorana syriaca* (17.4%), and *Adonis palaestina* (16.77%).

*Psidium guajava* (PG) leaf extract exerted an anti-diabetic and antihyperlipidemic effect by significantly reducing HSL activity in the liver and adipose tissue, improving serum lipid profile, reducing triglycerides, total cholesterol, and LDL-cholesterol levels, and increasing HDL-cholesterol levels, as well as increasing glycogen storage [23].

Culturing the *Streptomyces* species, DSM 13381, produces potent HSL inhibitors (IC_50_ values in the nanomolar range). These inhibitors are known as Cyclipostins and were isolated from the mycelium of *Streptomyces* species. Cyclipostins are neutral cyclic enol phosphate esters with an additional *γ*-lactone ring and have physicochemical properties such as triglycerides [85].

## 7. Conclusions

Hormone-sensitive lipase (HSL) is a crucial enzyme that releases fatty acids and glycerol from adipocyte lipid stores that are hormonally regulated. HSL affects lipid metabolism, glucose homeostasis, and cell signaling.

HSL is a multitasking enzyme due to its several activities, including the release of FFAs in the adipose tissue and the breakdown of cholesterol ester for steroidogenesis in the adrenals and gonads. Furthermore, it is highly expressed in the testes and necessary for spermatogenesis.

The breakdown of fats stored in adipose tissue via lipolysis provides free fatty acids as an energy source. On the other hand, excess free fatty acids (high lipolytic rate) are linked with low insulin sensitivity (or insulin resistance).

Inhibition of HSL can improve insulin sensitivity and control of blood glucose in type 2 diabetes. Therefore, many HSL inhibitors (synthetic and natural) have been identified and could potentially develop into effective antidiabetic agents.

## Data Availability

Data associated with the manuscript are included within the manuscript.

## References

[1] Zimmermann R., Lass A., Haemmerle G., Zechner R. (2009). Fate of fat: the role of adipose triglyceride lipase in lipolysis. *Biochimica et Biophysica Acta, Molecular and Cell Biology of Lipids*.

[2] Lass A., Zimmermann R., Oberer M., Zechner R. (2011). Lipolysis – a highly regulated multi-enzyme complex mediates the catabolism of cellular fat stores. *Progress in Lipid Research*.

[3] Viscarra J. A., Ortiz R. M. (2013). Cellular mechanisms regulating fuel metabolism in mammals: role of adipose tissue and lipids during prolonged food deprivation. *Metabolism*.

[4] Mottillo E. P., Shen X. J., Granneman J. G. (2007). Role of hormone-sensitive lipase in *β*-adrenergic remodeling of white adipose tissue. *American Journal of Physiology - Endocrinology And Metabolism*.

[5] Yeaman S. J. (2004). Hormone-sensitive lipase - new roles for an old enzyme. *Biochemical Journal*.

[6] Langfort J., Ploug T., Ihlemann J., Holm C., Galbo H. (2000). Stimulation of hormone-sensitive lipase activity by contractions in rat skeletal muscle. *Biochemical Journal*.

[7] Lafontan M., Langin D. (2009). Lipolysis and lipid mobilization in human adipose tissue. *Progress in Lipid Research*.

[8] Zechner R., Zimmermann R., Eichmann T. (2012). Fat SIGNALS - lipases and lipolysis in lipid metabolism and signaling. *Cell Metabolism*.

[9] Albert J. S., Yerges-Armstrong L. M., Horenstein R. B. (2014). Null mutation in hormone-sensitive lipase gene and risk of type 2 diabetes. *New England Journal of Medicine*.

[10] Savage D. B., Petersen K. F., Shulman G. I. (2007). Disordered lipid metabolism and the pathogenesis of insulin resistance. *Physiological Reviews*.

[11] Abu Farha R., Bustanji Y., Al-Hiari Y., Al-Qirim T., Abu Shiekha G., Albashiti R. (2016). Lipid lowering activity of novel N-(benzoylphenyl) pyridine-3-carboxamide derivatives in Triton WR-1339-induced hyperlipidemic rats. *Journal of Enzyme Inhibition and Medicinal Chemistry*.

[12] Zimmermann R., Haemmerle G., Wagner E. M., Strauss J. G., Kratky D., Zechner R. (2003). Decreased fatty acid esterification compensates for the reduced lipolytic activity in hormone-sensitive lipase-deficient white adipose tissue. *Journal of Lipid Research*.

[13] Shattat G. F. (2014). A review article on hyperlipidemia: types, treatments and new drug targets. *Biomedical and Pharmacology Journal*.

[14] Samuel V. T., Petersen K. F., Shulman G. I. (2010). Lipid-induced insulin resistance: unravelling the mechanism. *The Lancet*.

[15] Slee D. H., Bhat A. S., Nguyen T. N. (2003). Pyrrolopyrazinedione-based inhibitors of human hormone-sensitive lipase. *Journal of Medicinal Chemistry*.

[16] de Jong J. C., Sørensen L. G., Tornqvist H., Jacobsen P. (2004). Carbazates as potent inhibitors of hormone-sensitive lipase. *Bioorganic & Medicinal Chemistry Letters*.

[17] Ebdrup S., Sørensen L. G., Olsen O. H., Jacobsen P. (2004). Synthesis and Structure−Activity relationship for a novel class of potent and selective carbamoyl-triazole based inhibitors of hormone sensitive lipase. *Journal of Medicinal Chemistry*.

[18] Lowe D. B., et al e. a. (2004). *In vitro* SAR of (5-(2H)-Isoxazolonyl) ureas, potent inhibitors of hormone-sensitive lipase. *ChemInform*.

[19] Ebdrup S., Jacobsen P., Farrington A. D., Vedsø P. (2005). Structure–activity relationship for aryl and heteroaryl boronic acid inhibitors of hormone-sensitive lipase. *Bioorganic & Medicinal Chemistry*.

[20] Claus T. H., Lowe D. B., Liang Y. (2005). Specific inhibition of hormone-sensitive lipase improves lipid profile while reducing plasma glucose. *Journal of Pharmacology and Experimental Therapeutics*.

[21] Bustanji Y., Moulay A., Hudaib M. (2010). Hormone sensitive lipase inhibition by selected medicinal plants. *Journal of Medicinal Plants Research*.

[22] Ogiyama T., Yamaguchi M., Kurikawa N. (2017). Identification of a novel hormone sensitive lipase inhibitor with a reduced potential of reactive metabolites formation. *Bioorganic & Medicinal Chemistry*.

[23] Tella T., Masola B., Mukaratirwa S. (2019). The effect of *Psidium guajava* aqueous leaf extract on liver glycogen enzymes, hormone sensitive lipase and serum lipid profile in diabetic rats. *Biomedicine & Pharmacotherapy*.

[24] Østerlund T. (2001). Structure-function relationships of hormone-sensitive lipase. *European Journal of Biochemistry*.

[25] Langin D., Laurell H., Holst L. S., Belfrage P., Holm C. (1993). Gene organization and primary structure of human hormone-sensitive lipase: possible significance of a sequence homology with a lipase of Moraxella TA144, an antarctic bacterium. *Proceedings of the National Academy of Sciences*.

[26] Krintel C., Klint C., Lindvall H., Mörgelin M., Holm C. (2010). Quaternary structure and enzymological properties of the different hormone-sensitive lipase (HSL) isoforms. *PLoS One*.

[27] Smith G. M., Garton A. J., Aitken A., Yeaman S. J. (1996). Evidence for a multi-domain structure for hormone-sensitive lipase. *FEBS Letters*.

[28] Smith A. J., Sanders M. A., Juhlmann B. E., Hertzel A. V., Bernlohr D. A. (2008). Mapping of the hormone-sensitive lipase binding site on the adipocyte fatty acid-binding protein (AFABP). *Journal of Biological Chemistry*.

[29] Jenkins-Kruchten A. E., Bennaars-Eiden A., Ross J. R., Shen W. J., Kraemer F. B., Bernlohr D. A. (2003). Fatty acid-binding protein-hormone-sensitive lipase interaction. *Journal of Biological Chemistry*.

[30] Østerlund T., Contreras J. A., Holm C. (1997). Identification of essential aspartic acid and histidine residues of hormone-sensitive lipase: apparent residues of the catalytic triad. *FEBS Letters*.

[31] Watt M., Steinberg G. (2008). Regulation and function of triacylglycerol lipases in cellular metabolism. *Biochemical Journal*.

[32] Holm C., Kirchgessner T. G., Svenson K. L. (1988). Hormone-sensitive lipase: sequence, expression, and chromosomal localization to 19 cent-q13.3. *Science*.

[33] Lampidonis A. D., Rogdakis E., Voutsinas G. E., Stravopodis D. J. (2011). The resurgence of Hormone-Sensitive Lipase (HSL) in mammalian lipolysis. *Gene*.

[34] Grober J., Laurell H., Blaise R. (1997). Characterization of the promoter of human adipocyte hormone-sensitive lipase. *Biochemical Journal*.

[35] Stenson Holst L., Langin D., Mulder H. (1996). Molecular cloning, genomic organization, and expression of a testicular isoform of hormone-sensitive lipase. *Genomics*.

[36] Mulder H., Holst L. S., Svensson H. (1999). Hormone-sensitive lipase, the rate-limiting enzyme in triglyceride hydrolysis, is expressed and active in beta-cells. *Diabetes*.

[37] Laurin N. N., Wang S. P., Mitchell G. A. (2000). The hormone-sensitive lipase gene is transcribed from at least five alternative first exons in mouse adipose tissue. *Mammalian Genome*.

[38] Mairal A., Melaine N., Laurell H. (2002). Characterization of a novel testicular form of human hormone-sensitive lipase. *Biochemical and Biophysical Research Communications*.

[39] Fredrikson G., Stralfors P., Nilsson N. O., Belfrage P. (1981). Hormone-sensitive lipase of rat adipose tissue: purification and some properties. *Journal of Biological Chemistry*.

[40] Lee F. T., Adams J. B., Garton A. J., Yeaman S. J. (1988). Hormone-sensitive lipase is involved in the hydrolysis of lipoidal derivatives of estrogens and other steroid hormones. *Biochimica et Biophysica Acta (BBA) - Lipids and Lipid Metabolism*.

[41] Raclot T., Holm C., Langin D. (2001). Fatty acid specificity of hormone-sensitive lipase: implication in the selective hydrolysis of triacylglycerols. *Journal of Lipid Research*.

[42] Frühbeck G., Méndez-Giménez L., Fernández-Formoso J. A., Fernández S., Rodríguez A. (2014). Regulation of adipocyte lipolysis. *Nutrition Research Reviews*.

[43] Holm C., Østerlund T., Laurell H., Contreras J. A. (2000). Molecular mechanisms regulating hormone-sensitive lipase and lipolysis. *Annual Review of Nutrition*.

[44] Zimmermann R., Strauss J. G., Haemmerle G. (2004). Fat mobilization in adipose tissue is promoted by adipose triglyceride lipase. *Science*.

[45] Zechner R., Zimmermann R., Eichmann T. O. (2012). FAT SIGNALS-lipases and lipolysis in lipid metabolism and signaling. *Cell Metabolism*.

[46] Sztalryd C., Xu G., Dorward H. (2003). Perilipin A is essential for the translocation of hormone-sensitive lipase during lipolytic activation. *Journal of Cell Biology*.

[47] Londos C., Brasaemle D. L., Schultz C. J. (1999). On the control of lipolysis in adipocytes. *Annals of the New York Academy of Sciences*.

[48] Holm C., Langin D., Manganiello V. C., Belfrage P., Degerman E. (1997). Regulation of hormone-sensitive lipase activity in adipose tissue. *Methods in Enzymology*.

[49] DiPilato L. M., Ahmad F., Harms M., Seale P., Manganiello V., Birnbaum M. J. (2015). The role of PDE3B phosphorylation in the inhibition of lipolysis by insulin. *Molecular and Cellular Biology*.

[50] Rahn T., Rönnstrand L., Leroy M. J. (1996). Identification of the site in the cGMP-inhibited phosphodiesterase phosphorylated in adipocytes in response to insulin and isoproterenol. *Journal of Biological Chemistry*.

[51] Wijkander J., Landström T. R., Manganiello V., Belfrage P., Degerman E. (1998). Insulin-induced phosphorylation and activation of phosphodiesterase 3B in rat adipocytes: possible role for protein kinase B but not mitogen-activated protein kinase or p70 S6 kinase. *Endocrinology*.

[52] Elks M. L., Manganiello V. C. (1985). Antilipolytic action of insulin: role of cAMP phosphodiesterase activation. *Endocrinology*.

[53] Eriksson H., Ridderstråle M., Degerman E. (1995). *Biochimica et Biophysica Acta*.

[54] Girousse A., Langin D. (2011). Adipocyte lipases and lipid droplet-associated proteins: insight from transgenic mouse models. *International Journal of Obesity*.

[55] Egan J. J., Greenberg A. S., Chang M.-K., Londos C. (1990). *Journal of Biological Chemistry*.

[56] Smith U., Carvalho E., Mosialou E., Beguinot F., Formisano P., Rondinone C. (2000). PKB inhibition prevents the stimulatory effect of insulin on glucose transport and protein translocation but not the antilipolytic effect in rat adipocytes. *Biochemical and Biophysical Research Communications*.

[57] Kraemer F. B., Shen W. J. (2002). Hormone-sensitive lipase. *The Journal of Lipid Research*.

[58] Shen W.-J., Sridhar K., Bernlohr D. A., Kraemer F. B. (1999). Interaction of rat hormone-sensitive lipase with adipocyte lipid binding protein. *Proceedings of the National Academy of Sciences of the United States of America*.

[59] Syu L. J., Saltiel A. R. (1999). Lipotransin: a novel docking protein for hormone-sensitive lipase. *Molecular Cell*.

[60] White U., Ravussin E. (2018). Dynamics of adipose tissue turnover in human metabolic health and disease. *Diabetologia*.

[61] Cinti S. (2012). The adipose organ at a glance. *Disease Models & Mechanisms*.

[62] Bjørndal B., Burri L., Staalesen V., Skorve J., Berge R. K. (2011). Different adipose depots: their role in the development of metabolic syndrome and mitochondrial response to hypolipidemic agents. *Journal of Obesity*.

[63] Ström K., Gundersen T. E., Hansson O. (2009). Hormone-sensitive lipase (HSL) is also a retinyl ester hydrolase: evidence from mice lacking HSL. *The FASEB Journal*.

[64] Guilherme A., Virbasius J. V., Puri V., Czech M. P. (2008). Adipocyte dysfunctions linking obesity to insulin resistance and type 2 diabetes. *Nature Reviews Molecular Cell Biology*.

[65] Michalik L. (2006). Involvement of PPAR nuclear receptors in tissue injury and wound repair. *Journal of Clinical Investigation*.

[66] Zechner R., Kienesberger P. C., Haemmerle G., Zimmermann R., Lass A. (2009). Adipose triglyceride lipase and the lipolytic catabolism of cellular fat stores. *Journal of Lipid Research*.

[67] Alsted T. J., Ploug T., Prats C. (2013). Contraction-induced lipolysis is not impaired by inhibition of hormone-sensitive lipase in skeletal muscle. *The Journal of Physiology*.

[68] Roepstorff C., Vistisen B., Donsmark M. (2004). Regulation of hormone-sensitive lipase activity and Ser563and Ser565 phosphorylation in human skeletal muscle during exercise. *The Journal of Physiology*.

[69] Ueno M., Suzuki J., Zenimaru Y. (2008). Cardiac overexpression of hormone-sensitive lipase inhibits myocardial steatosis and fibrosis in streptozotocin diabetic mice. *American Journal of Physiology - Endocrinology And Metabolism*.

[70] Suzuki J., Ueno M., Uno M. (2009). Effects of hormone-sensitive lipase disruption on cardiac energy metabolism in response to fasting and refeeding. *American Journal of Physiology - Endocrinology And Metabolism*.

[71] Li H., Brochu M., Wang S. P. (2002). Hormone-sensitive lipase deficiency in mice causes lipid storage in the adrenal cortex and impaired corticosterone response to corticotropin stimulation. *Endocrinology*.

[72] Shen W. J., Patel S., Natu V. (2003). Interaction of hormone-sensitive lipase with steroidogeneic acute regulatory protein. *Journal of Biological Chemistry*.

[73] Arenas M. I., Lobo M. V., Caso E., Huerta L., Paniagua R., Martín-Hidalgo M. A. (2004). Normal and pathological human testes express hormone-sensitive lipase and the lipid receptors CLA-1/SR-BI and CD36. *Human Pathology*.

[74] Vallet-Erdtmann V., Tavernier G., Contreras J. A. (2004). The testicular form of hormone-sensitive lipase HSL tes confers rescue of male infertility in HSL-deficient mice. *Journal of Biological Chemistry*.

[75] Blaise R., Guillaudeux T., Tavernier G. (2001). Testis hormone-sensitive lipase expression in spermatids is governed by a short promoter in transgenic mice. *Journal of Biological Chemistry*.

[76] Casado M. E., Huerta L., Ortiz A. I. (2012). HSL-knockout mouse testis exhibits class B scavenger receptor up regulation and disrupted lipid raft micro-domains. *Journal of Lipid Research*.

[77] Osuga J. i., Ishibashi S., Oka T. (2000). Targeted disruption of hormone-sensitive lipase results in male sterility and adipocyte hypertrophy, but not in obesity. *Proceedings of the National Academy of Sciences*.

[78] Chung S., Wang S. P., Pan L., Mitchell G., Trasler J., Hermo L. (2001). Infertility and testicular defects in hormone-sensitive lipase-deficient mice. *Endocrinology*.

[79] Lindvall H., Nevsten P., Ström K. (2004). A novel hormone-sensitive lipase isoform expressed in pancreatic *β*-cells. *Journal of Biological Chemistry*.

[80] Sorhede Winzell M., Svensson H., Enerback S. (2003). Pancreatic -cell lipotoxicity induced by overexpression of hormone-sensitive lipase. *Diabetes*.

[81] Roduit R., Masiello P., Wang S. P., Li H., Mitchell G. A., Prentki M. (2001). A role for hormone-sensitive lipase in glucose-stimulated insulin secretion: a study in hormone-sensitivelipase-deficient mice. *Diabetes*.

[82] Khan A., Narangoda S., Ahren B., Holm C., Sundler F., Efendic S. (2001). Long-term leptin treatment of ob/ob mice improves glucose-induced insulin secretion. *International Journal of Obesity*.

[83] Arner P., Langin D. (2014). Lipolysis in lipid turnover, cancer cachexia, and obesity-induced insulin resistance. *Trends in Endocrinology and Metabolism*.

[84] Schweiger M., Romauch M., Schreiber R. (2017). Pharmacological inhibition of adipose triglyceride lipase corrects high-fat diet-induced insulin resistance and hepatosteatosis in mice. *Nature Communications*.

[85] Wink J., Schmidt F. R., Seibert G., Aretz W. (2010). ChemInform abstract: Cyclipostins: novel hormone-sensitive lipase inhibitors from *Streptomyces* sp. DSM 13381. Part 1. Taxonomic studies of the producer microorganism and fermentation results. *ChemInform*.

[86] Bustanji Y., Issa A., Mohammad M. (2011). Inhibition of hormone sensitive lipase and pancreatic lipase by *Rosmarinus officinalis* extract and selected phenolic constituents. *Journal of Medicinal Plants Research*.

[87] Ebdrup S., Refsgaard H. H. F., Fledelius C., Jacobsen P. (2007). Synthesis and Structure−Activity relationship for a novel class of potent and selective carbamate-based inhibitors of hormone selective lipase with acute *in vivo* antilipolytic effects. *Journal of Medicinal Chemistry*.

[88] Ogiyama T., Yamaguchi M., Kurikawa N. (2016). Identification of a novel boronic acid as a potent, selective, and orally active hormone sensitive lipase inhibitor. *Bioorganic & Medicinal Chemistry*.

[89] He C., Wan H. (2018). Drug metabolism and metabolite safety assessment in drug discovery and development. *Expert Opinion on Drug Metabolism and Toxicology*.

[90] Ben Ali Y., Verger R., Carrière F., Petry S., Muller G., Abousalham A. (2012). The molecular mechanism of human hormone-sensitive lipase inhibition by substituted 3-phenyl-5-alkoxy-1, 3, 4-oxadiazol-2-ones. *Biochimie*.

[91] Bolsoni-Lopes A., Alonso-Vale M. I. C. (2015). Lipolysis and lipases in white adipose tissue: an update. *Archives of Endocrinology and Metabolism*.

[92] Dahabiyeh L. A., Bustanji Y., Taha M. O. (2019). The herbicide quinclorac as potent lipase inhibitor: discovery via virtual screening and *in vitro/in vivo* validation. *Chemical Biology & Drug Design*.

